# Flexible skill-based control for robot cells in manufacturing

**DOI:** 10.3389/frobt.2022.1014476

**Published:** 2022-09-29

**Authors:** Torben Wiese, Johannes Abicht, Christian Friedrich, Arvid Hellmich, Steffen Ihlenfeldt

**Affiliations:** IIoT Controls and Technical Cybernetics, Fraunhofer Institute for Machine Tools and Forming Technology, Dresden, Germany

**Keywords:** skill-based control, flexible control systems, robot cells, modular automation, robot skills

## Abstract

Decreasing batch sizes lead to an increasing demand for flexible automation systems in manufacturing industries. Robot cells are one solution for automating manufacturing tasks more flexibly. Besides the ongoing unifications in the hardware components, the controllers are still programmed application specifically and non-uniform. Only specialized experts can reconfigure and reprogram the controllers when process changes occur. To provide a more flexible control, this paper presents a new method for programming flexible skill-based controls for robot cells. In comparison to the common programming in logic controllers, operators independently adapt and expand the automated process sequence without modifying the controller code. For a high flexibility, the paper summarizes the software requirements in terms of an extensibility, flexible usability, configurability, and reusability of the control. Therefore, the skill-based control introduces a modularization of the assets in the control and parameterizable skills as abstract template class methodically. An orchestration system is used to call the skills with the corresponding parameter set and combine them into automated process sequences. A mobile flexible robot cell is used for the validation of the skill-based control architecture. Finally, the main benefits and limitations of the concept are discussed and future challenges of flexible skill-based controls for robot cells are provided.

## 1 Introduction: Current challenges of controls for robot cells

In the manufacturing industry, robots offer a productive and flexible solution to automate manufacturing processes. Due to their serial design and uniform mechanical interfaces, robots are used as manipulators for variable, repetitive and high-precision tasks (Arents and Greitans, 2022). Typical applications include basic handling applications (e.g., for parts, pallets) as well as more complex processes, such as welding or the assembly of parts (Siciliano and Khatib, 2016). To add the necessary skills to the robot, assets, like grippers, sensors, and actuators, are applied to form task-specific static or mobile robot cells (Lienenluke et al., 2018 - 2018; Wojtynek et al., 2019; Sanneman et al., 2020).

In mass production, e.g., the automotive industry, such robotic cells are common. However, the increasing number of product variants requires more flexible robot cells in hardware and software to adapt them to the current processes (Dorofeev and Wenger, 2019 - 2019; Saukkoriipi et al., 2020). Therefore, the retooling and reconfiguration of robot cells are key challenges for the current research (Jörgen Frohm et al., 2006; T. Dietz, 2012). In hardware, there exist standardized mechanical, electrical, and data interfaces for modular assets to enable flexibility (Radanovic et al., 2021 - 2021). This is known as Plug and Produce concept (Pfrommer et al., 2015; Wojtynek et al., 2019; Falkowski et al., 2020). In software, configuring and teaching robot controllers (RC) and programmable logic controllers (PLC) is still a non-uniform, time-consuming and skill-demanding bottleneck (Sanneman et al., 2020; Zhou et al., 2020). An expert with control programming knowledge is necessary to reconfigure the robot cell PLC. Operators with basic process knowledge are not able to adjust the control software effortless. Therefore, programming experts must define all possible process changes in software that limits the flexibility to serval static case clauses (Deutschmann et al., 2020). In addition, the monolithic programming of controllers, non-uniform interfaces and static graphical user interfaces further decrease the software flexibility in robot cell controls. Hence, the standardization of communication interfaces and more abstract, task-oriented programming becomes very important to increase the software flexibility of robot cells (Saukkoriipi et al., 2020; Heimann and Guhl, 2020 - 2020; Sanneman et al., 2020). As a result, the following requirements for flexible controls of robot cells are defined:

• Extensibility: To be able to adapt a robot cell to changing processes, it must be possible to extend it with adapted or new assets to be able to use their manufacturing functions for the process sequences. Besides the hardware connectors, the extensibility must be ensured in terms of software. The control architecture, therefore, must deal with real-time capabilities, computing power, and communication interfaces of the control systems of the assets.
• Flexible usability: The individual manufacturing functions of the assets must be flexibly usable for the operator. To ensure flexibility, each asset should provide its functions independently of other assets to combine them independently into sequences. By defining automated sequences, the operator assembles the functions into more complex process steps.
• Configurability: The control of flexible robot cells must enable a configurability of the automated sequences to the operator based on his detailed manufacturing process knowledge. The individual functions of the assets must be configurable *via* changeable parameters to be able to adapt them to specific process steps. This allows the operator to configurate sequences with differently parameterized function calls of the assets without having control programming expertise.
• Reusability: Already defined functions and sequences should be reusable to reduce reprogramming and increase commissioning time. In this way, the operator can access already working process steps and generate new process sequences without having to adapt individual functions. Process steps can also be exchanged and reused between different robot cells with the same functionalities.

One promising approach to fulfilling the requirements of a flexible robot cell control is the skill-based control architecture (SBC) (Dorofeev and Wenger, 2019 - 2019). The SBC uses an abstraction concept by composing single manufacturing tasks through parameterizable, component-specific skills. An orchestration layer manages the task-specific arrangement and call of the skills. Each component offers its skills *via* uniform software interfaces for data communication (Pfrommer et al., 2014 - 2014). Beside other self-describing component modelling approaches according to the Industry-4.0 concept, OPC-UA is commonly used as a universal communication interface (Zimmermann et al., 2019 - 2019). Not only for combining skills but also for unified and flexible multi-system orchestration, SBC together with OPC-UA enables immense benefits in software implementation and reconfiguration (Profanter et al., 2019). In SkillPro (Brandenbourger and Durand, 2018 - 2018), RAZER (Steinmetz et al., 2018), and other projects (Saukkoriipi et al., 2020), the successful implementation has been validated. The VDI/VDE has published the first standardizations of skills in a guideline in field of process industry. This guideline focuses on modularization, the service interfaces, parametrization, state machines, and behavior models (Deutsches Institut fur Normung, 2020). Today, main deficits are:

• Despite the increasing efforts in standardization and tests, SBCs are not widely used in the manufacturing industry, compared to established monolithically programmed control systems.
• Unified models for components and skills for manufacturing processes are missing (Malakuti et al., 2018 - 2018).
• Control systems are only programmed by experts. New concepts need to simplify control programming for non-experts (Pedersen et al., 2016).

This paper presents an approach of a skill-based control for flexible robot cells for manufacturing. Therefore, the approach proposes a control architecture that fulfils the requirements of extensibility, flexible usability, configurability, and reusability. The verification of requirements is analysed on a flexible robot cell for machine tool automation.

## 2 Method: Development of a skill-based control for flexible robot cells

The development of the SBC divides into three methodical subgoals of the control software. The order of the subgoals represents the workflow during implementation on the robot cell controller. First, all assets are modularized, followed by assigning the functions of the assets to the modules as parameterizable skills. Finally, the SBC is extended by an orchestration system of the skills to automate the skills into process sequences. In the following section, the subgoals are presented.

Modularization starts by dividing the assets of a robot cell into functionally separable subsystems that work and are controlled independently. Therefore, object-oriented programming ensures uniform states and interfaces. In the SBC, a superclass as a template for a unified asset module is defined. New asset modules are thus created by inheritance of the template module. This approach enables consistent handling, monitoring, state control, and error management of all the different modules in a robot cell.

**FIGURE 1:**
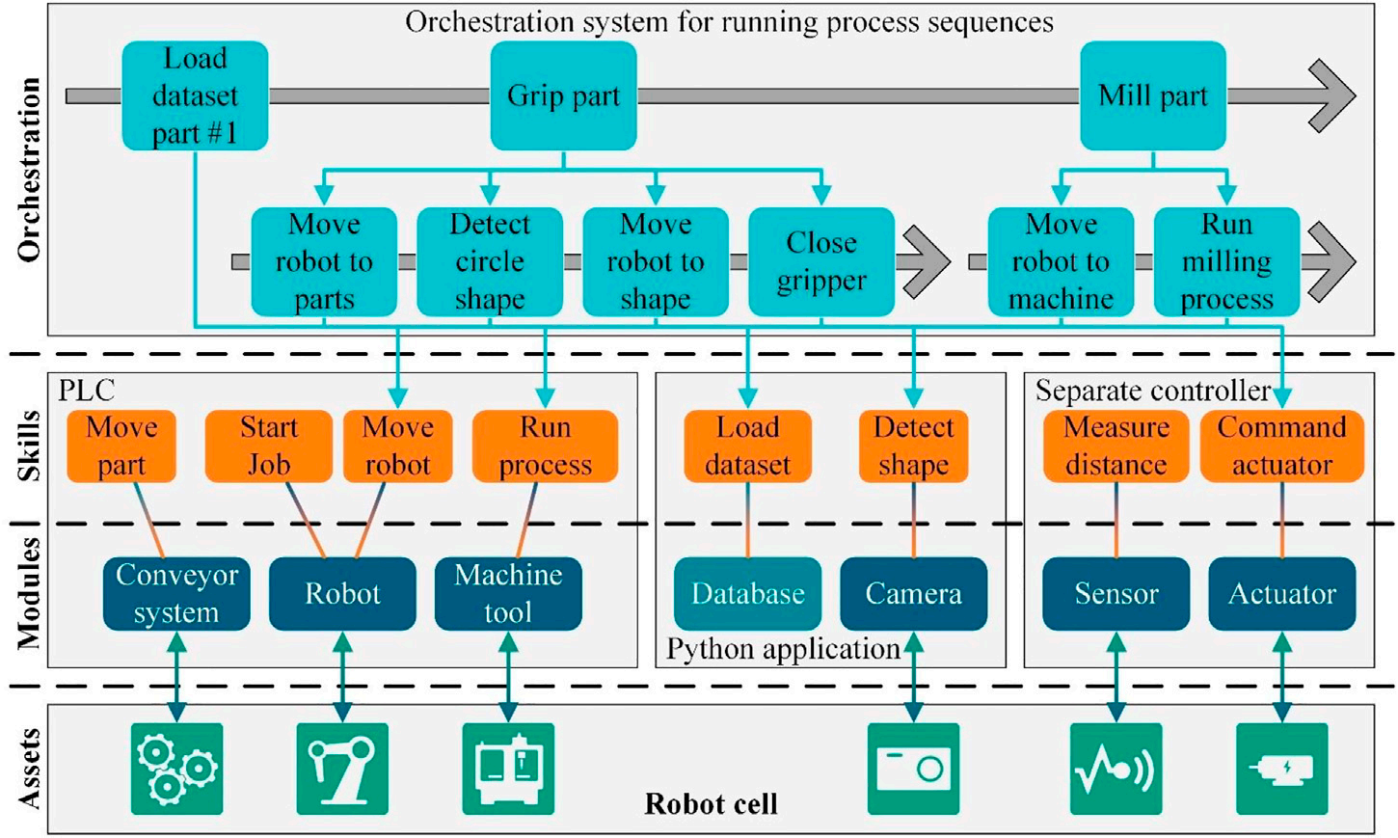
Concept of a robot cell automation by a skill-based control architecture.

Figure 1 illustrates the linking of assets and their corresponding software modules in different control systems of the robot cell. Depending on the controller architecture, module controllers can also run in different controllers or applications as long as the communication and linking with the module handling is realized. Beneficially, the specific requirements for asset controls in terms of necessary real-time capability, hardware connectivity, and computing performance can be considered and implemented individually. This allows the decentralized allocation of control tasks to performance-specific, separated controllers which reduces hardware costs. Modules can also be arranged hierarchically at different levels and consist of different sub-modules to consider the physical linking of assets in the controller. The communication between the modules of different controllers is realized *via* various manufacturer- and programming language-independent interfaces, such as OPC-UA. The modularization of controls for all assets enables the extensibility of the robot cell at the software level. New assets and their control modules can be integrated *via* uniform interfaces through template inheritance.

To provide the asset functions, such as “move” of a robot or “close” of a gripper, parameterizable skills for modules are defined, as shown in Figure 1. In programming, the bottom-up approach can be used to implement the available functions for each asset as skills in the module control. The control programmer should implement not only the asset functions that are necessary for the overall automation solution, but also the functions that the asset can perform independently of other assets. This guarantees the flexible usability of all functionalities of the assets. Another important aspect is the possibility to parameterize the skills to adapt the individual asset functions to different process tasks. For example, the target position can be specified as a parameter for a robot movement to enable configurability by the operator.

Modules and their skills represent the basic functions of the various assets in a robot cell. To combine them into an automated process sequence, e.g., a machine tool tending process, an orchestration system in the master controller is required. The orchestration system defines, parameterizes, and controls the process sequence. Due to the modularization, the orchestration system can communicate with all available module skills using different communication interfaces like both vendor-specific and vendor-independent ones. Complex process sequences can be configured by parameterizing and combining skills into reusable steps, whereby the operator can flexibly change individual parameters of skills or entire steps at any time. When creating automated process sequences, the top-down approach is ideal to generate detailed sub-steps in various abstraction levels. The operator can use his detailed knowledge of the manufacturing process and first create abstract process steps, which are then specified in further sub-steps and finally call up the individual skills with configured parameters. The orchestration level in Figure 1 shows an exemplary process flow with abstract steps, which in turn contain more concrete sub-steps. Reusability is ensured by storing the sequences and steps in data lists that contain the information about the skill connection and associated parameters. With a suitable human machine interface (HMI), the operator can configure and parameterize the process sequences without programming, which means that no knowledge of programming in the controls is required.

**FIGURE 2:**
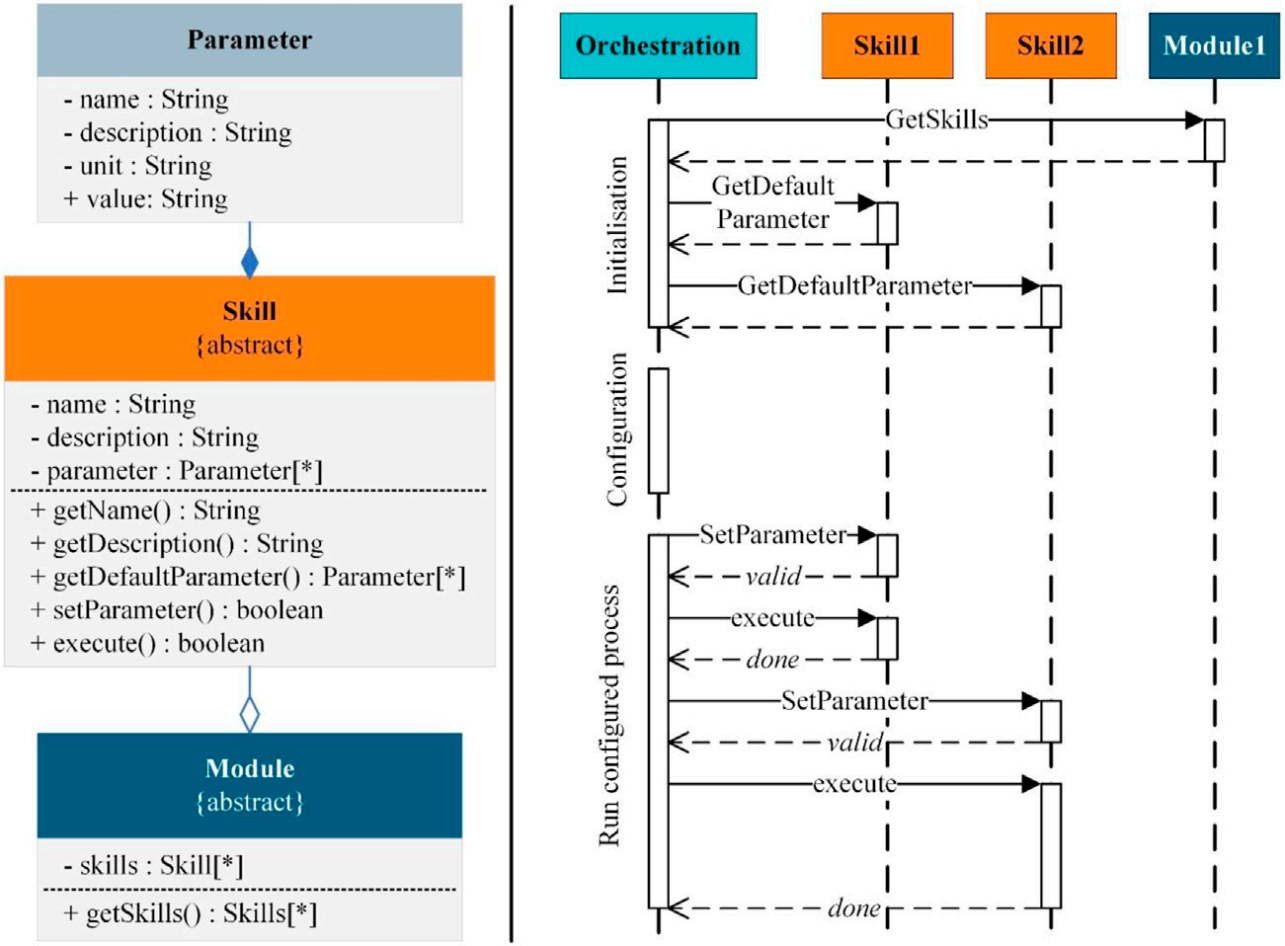
Class diagram for the implementation of parameterizable skills in modules (left) and sequence diagram for their orchestration (right).

For the communication between skills and the orchestration system or operator, each skill provides the necessary meta information about itself, such as its name, description, and the associated asset as well as the information about its adjustable parameters. The left side of Figure 2 presents an exemplary class diagram of the abstract skill class with the necessary parameters and methods for implementation in control systems. Every skill deriving from the abstract class can be connected in the same way, by accessing properties and using methods providing the information and control options. To be able to parameterize the skills uniformly, self-describing parameters using a generalized definition structure are introduced. The orchestration system can use the methods to retrieve the default parameters, set new parameter values as well as to execute the skill that triggers the associated function of the asset with the specified parameters. After the configuration and parameterization of a process sequence, the steps can be processed *via* the unified interfaces to the skill and thus an automated flow can be accomplished. The right side of Figure 2 shows a sequence diagram for the exemplary execution of a process sequence by an orchestration system.

## 3 Results: Verification of the flexibility of the skill-based control on a mobile robot cell

**FIGURE 3:**
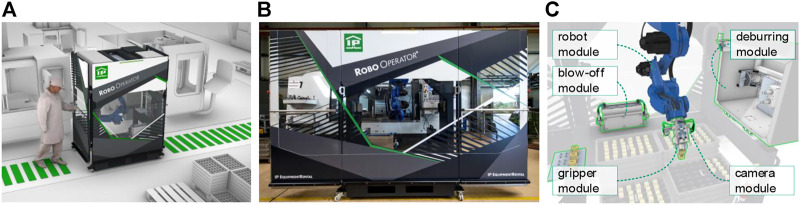
**(A–C)**: The Robo Operator ^©^ automates operator tasks on machine tools by skills.

To evaluate the proposed SBC in terms of the flexibility requirements, a test platform has been selected. Therefore, the SBC was implemented on the Robo Operator^©^ (RO) using a TwinCAT PLC. The RO is a mobile robot cell, as shown in Figure 3 (A-C), that was developed in a research project between Fraunhofer IWU and Industrie-Partner GmbH (Abicht et al., 2021). As flexible automation solution, it automates tasks of operators on machine tools. Therefore, an industrial robot (Yaskawa GP12) with a 2-jaw gripper and a smart camera (Intel Realsense D435i) enable the RO to move parts, to open and close doors, and to start the machine tool by control panel interaction. The smart camera provides the position information of all relevant objects. Applicable asset-modules, e.g., deburring or blow-off modules, extend the workflow with new skills for the manufacturing process, see Figure 3C.

**FIGURE 4:**
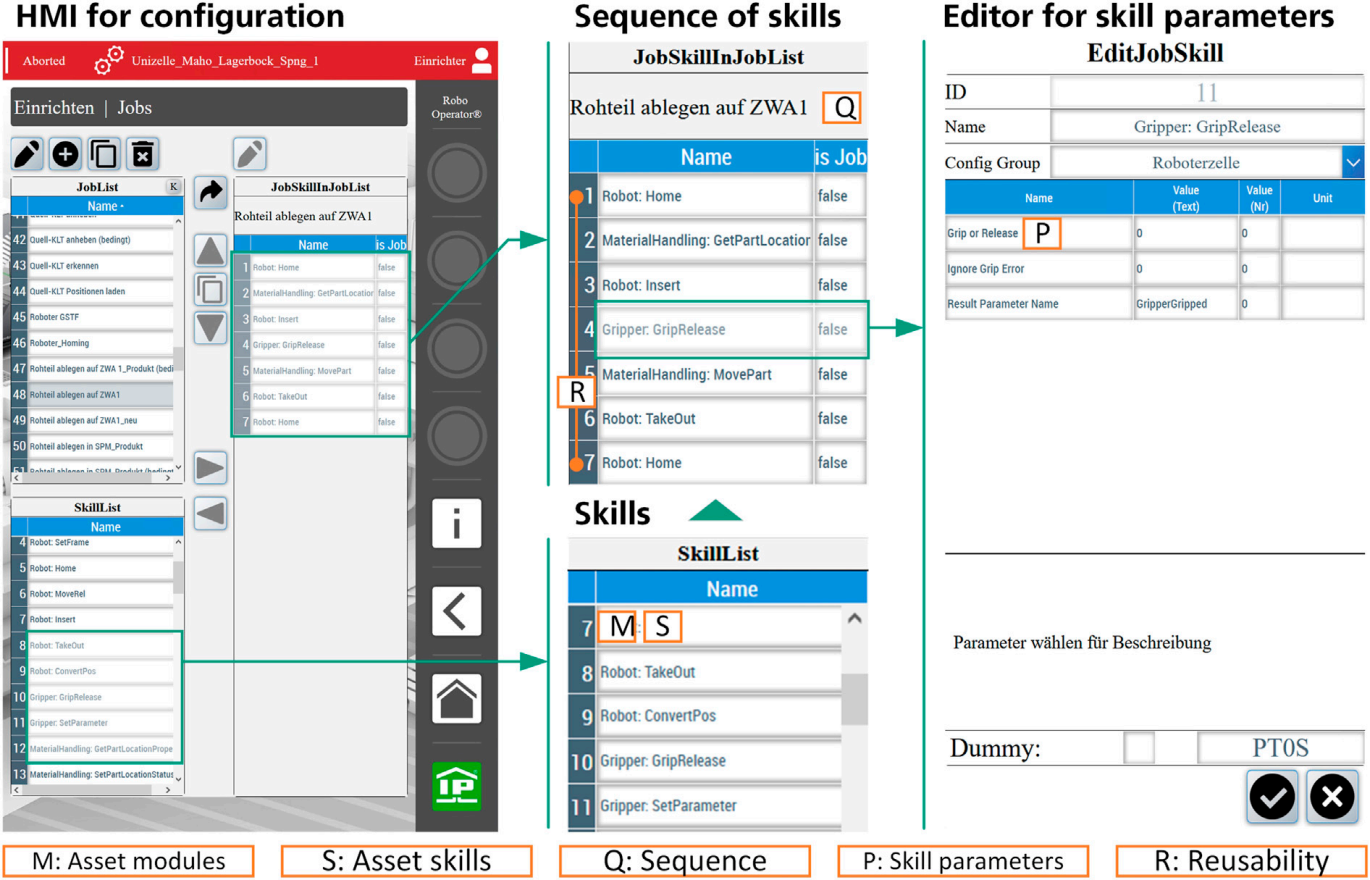
Composition of the module skills to sequences and skill parameterization through the configuration HMI.

With the given structure, the RO represents a flexible robot cell in manufacturing. The flexibility of hardware is reached by standardized Han^®^ connectors and the capsuled design of the asset modules. In the following section, the paper analyses how the methods of the proposed SBC architecture realize the flexibility in the control. Therefore, the paper discusses the implementation based on the four aspects from chapter 1. The human machine interface (HMI) of the RO visualizes the achieved results, see Figure 4. At the HMI, operators configure the process through the composition of skills into sequences.

While implementing the SBC into the RO, all assets get an own functionally separated software module. As shown in Figure 4, e.g., the asset modules (M) “Robot” for the Yaskawa GP12 and “Gripper” for the 2-jaw gripper are programmed. Other modules, such as the camera, blow-off and, deburring module, could be created by inheritance of the template module easily to verify the extensibility of the software. Based on the template, other modules can be integrated into the process in this way. Due to the TwinCAT EtherCAT Hot-Connect functionality, the modules are initialized automatically when the corresponding assets are plugged by Han^®^ connectors. A state machine was implemented to manage the module states, such as Idle, Error, or Resetting, while automating the process. The communication between the modules is based on TwinCAT ADS (Automation Device Specification) as universal software interface.

The definition of skills for all asset modules guarantees the flexible usability of the software inside the master control system, see the asset skills (S) in Figure 4. To achieve a specific goal or sub-task of a manufacturing process, the skills are combined into a sequence of skills (Q). For example, the sequence “Place raw part on buffer plate” (in Figure 4: “Rohteil ablegen auf ZWA1”) places a gripped raw part on a buffer plate to determine the position of the part more accurately. Physically*,* the RO inserts the gripped part from a home position of the robot to the buffer plate position, opens the gripper and returns to the home position safely. In the HMI, the operator combines the skills Home, Insert, and TakeOut from the robot module with a GripRelease skill from the gripper module from the SkillList, as shown in Figure 4.

To use the module skills for different processes in a flexible way, they must fulfil the configurability requirement. In the skill editor in Figure 4, the operator adjusts the skills to the current requirements of the process by specific skill parameters (P). For example, operators must configure if the gripper should be opened or closed at a grip or release position. By inheriting the skill template class from Figure 2, the GripRelease-Skill is defined with the skill-specific parameters Grip or Release and additional parameters that configure the skill in the RO control to the current task without changing PLC code. The orchestration module executes every skill with the configured skill parameters while automating the complete process sequence.

**FIGURE 5:**
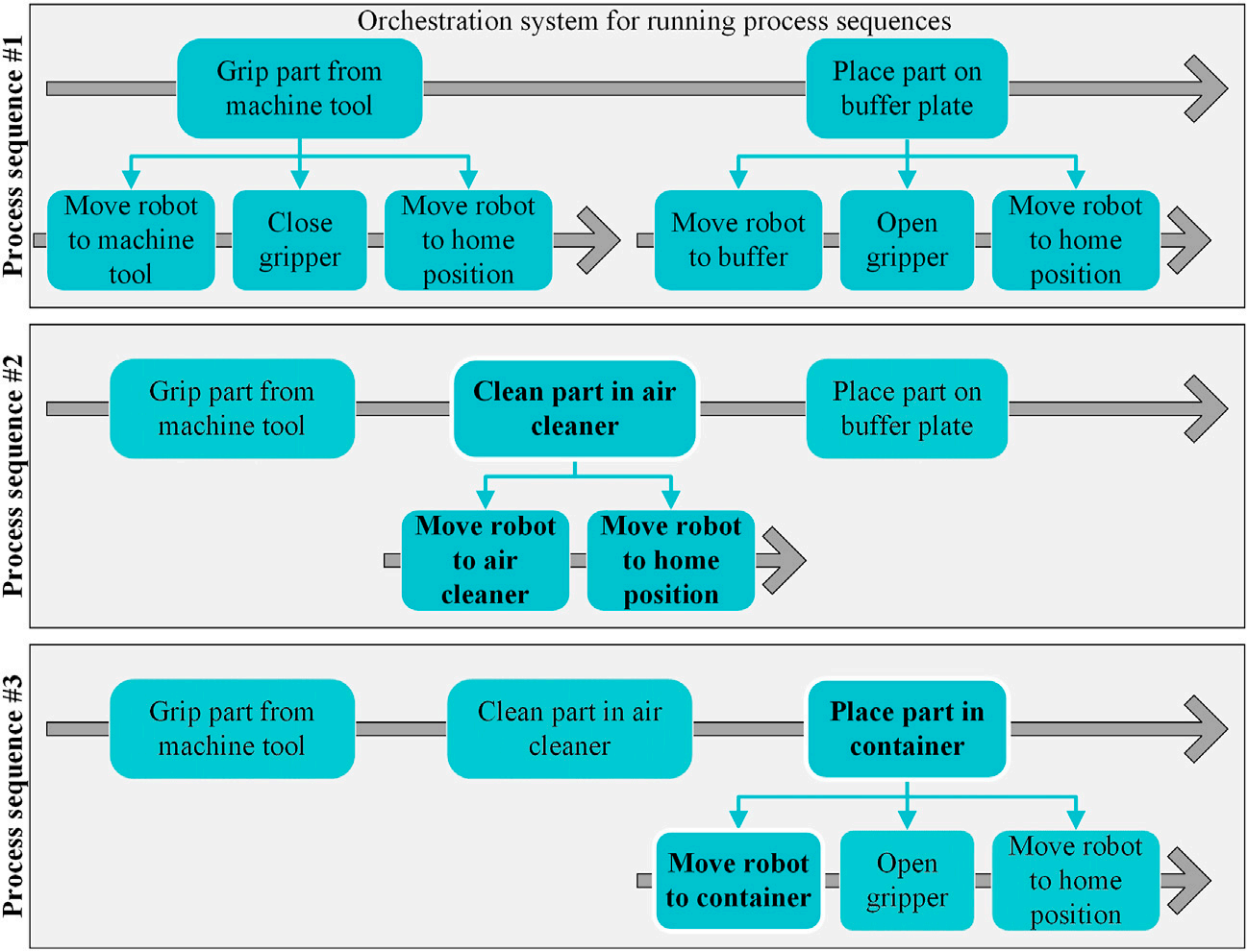
Example for modifying an automated process sequence of a robot cell with skill-based control.

Predefined sequences can also be used as steps in a higher-level sequence to reduce the configuration effort. This allows an operator without detailed knowledge of single sequence steps to configurate a sequence for the RO. For example, a simple process sequence automating the move of one part from a machine tool to a buffer plate, as shown by sequence #1 in Figure 5, can be extended with an intermediate step to clean the part with a blow-off module. For this, the operator only has to insert the necessary process step in the orchestration system, which contains the parameterized skills calls (see Sequence #2 in Figure 5). Furthermore, the operator can change the already existing step for placing the part by a new destination, such as a container. The operator can do this by adjusting the parameterization of the skill that makes the robot move to a specific position, in this case by changing the target position from “buffer” to “container” (see Sequence #3 in Figure 4).

The combination of parameterized skills and pre-defined sub-sequences to new sequences enables a high reusability. In Figure 4 the “Place raw part on buffer plate” sequence uses more than one Home-Skill (R). To save the configuration in the HMI, the SBC of the RO has an additional data base module to save and reload all the information about the skills and parameters as well as the sequences itself.

## 4 Conclusion

The present paper proposes a method for implementing a flexible control architecture in robot cells called skill-based control. In chapter 1, four requirements were defined for developing the method for implementing the skill-based control. Main reasons for developing this method are missing guidelines or unifications for flexible control architectures for manufacturing purposes and the wide range of programmer expertise levels that must be conducted.

Methodically, the skill-based controls consist of a software modularization of all assets, definition of capsuled asset functions in skills and an orchestration system for skill management and calling. Based on object-oriented programming, template classes have been implemented for the asset modules and skills. For an extension of an automation, the templates can be used to easily create specific modules. Thus, the communication structure of the modules is unified. As communication protocols, OPC-UA or similar universal manufacturer-independent standardizations are proposed. To adapt the skills to the current situation, each skill call be individualized by parameters defined by the skill developer.

The results of the paper show that the skill-based control fulfils all requirements of a flexible control for robot cells. For verification of the methods, the skill-based control was successfully implemented on a mobile robot cell. The implementation shows the fast programming through the reusability of the software components in the human machine interface. Furthermore, the programming of the software is reduced to the combination of skills and process steps to sequences on a non-programming-level that is potentially less time-consuming than static programming.

As the method was used to implement the skill-based control for the RO, it quickly becomes clear that the flexibility available to the operator depends primarily on the type and amount of provided skills and their parameters. For high flexibility, many skills and adjustable parameters are needed, requiring a longer development time. To reduce the resulting complexity, the operator must be offered predefined process steps that combine frequently used skill combinations and their parameters. Furthermore, dependencies between skills that may not be known to the operator must be represented in the orchestration system. Knowledge about the dependencies of the skills and process steps is crucial for the configuration of fault-free process sequences. Therefore, a critical development goal is to further reduce the expertise required in the use of skill-based controls mainly by expanding the orchestration system.

For further studies, the interoperability of the software modules on different master controllers and their corresponding programming languages must be conducted. Because the flexible usability, configurability and reusability of skills and sequences depends mainly on the usability of the HMI, so more research in HMI design and layout is necessary. Therefore, the intuitiveness, modularity, uniformity, security, and robustness must be considered. Finally, it must be researched how suitable the proposed skill-based control fits to larger production lines or matrix production systems.

## Data Availability

The original contributions presented in the study are included in the article/supplementary material, further inquiries can be directed to the corresponding author.
